# Standing wave effect and fractal structure in dislocation evolution

**DOI:** 10.1038/s41598-017-04257-9

**Published:** 2017-06-22

**Authors:** P. Li, Z. F. Zhang

**Affiliations:** 1 Shenyang National Laboratory for Materials Science, Institute of Metal Research, Chinese Academy of Sciences, 110016 Shenyang, China; 2 State Key Laboratory of Metastable Materials Science and Technology, Yanshan University, 066004 Qinhuangdao, China; 3 Institut für Werkstoffe, Ruhr-Universität Bochum, 44780 Bochum, Germany

## Abstract

Theoretical model required for the evolution of regular dislocation pattern should simultaneously take into account both static distribution and dynamic evolution of dislocation pattern. In principle, there exists a stable uniformly moving dislocation with both core and far field advancing at the same constant velocity, which suggests the existence of the traveling waves representing moving dislocation. Therefore, one new term “dislocation wave” is defined by simultaneously consisting of both an elastic wave and a dislocation in each wavefront. According to the standing wave effect, the edge dislocation segments capture mutually to form the periodic ladder structures at the nodes. These persistent slip band (PSB) ladders are not only self-organized but also self-similar dislocation patterns. The fractal dimension further reveals the intrinsic nature of crack initiation and propagation along slip bands and deformation bands.

## Introduction

The term “dislocation” is more established for line defects in crystals in the 1930s^1–3^, that is, imperfections in a regular periodic lattice geometry. Since then, these geometric topological defects become the most important deformation mode in crystal materials^4, 5^. The studies concerning these defects can be classified into two important aspects. One is the dislocation multiplication, which originates from the continuous movement of dislocation; the other is the dislocation interplay, which leads to different topological distribution of dislocations. The assembly and regular distribution of a large number of dislocations eventually evolve into all kinds of pattern structures, among which the most typical structure is persistent slip bands (PSBs). In 1956, Thompson *et al*.^6^ found that these PSBs reappeared at the old sites when the specimen was fatigued again after the previously formed slip bands had been polished away, which indicated that the microstructure in the bulk of PSBs is different from that of the surrounding matrix. Later, these PSBs were confirmed convincingly to be nor merely a surface but a bulk phenomenon by Laufer and Roberts^7^. Since PSBs consisted of the so-called wall or ladder structures^8^, the basic feature of PSB is determined to be the spatial periodically ordered structure with the alternate appearance of dislocation-rich and dislocation-poor regions^9^.

On the other hand, low-energy dislocation structure (LEDS) model and self-organized dislocation structure (SODS) model are two main theories to describe the formation of periodic dislocation patterns. The former focuses on analyzing the composition and distribution of static dislocation structures based on the Taylor-Nabarro lattice; the latter regards the dislocation patterning as an example of self organization in a system driven far from equilibrium. These theoretical models of dislocation evolution are still in their infancy, the main difficulty for further improving the above models to simultaneously take into account both static distribution and dynamic evolution of dislocation pattern^10, 11^. Although the existing dislocation theory can explain the properties of individual dislocations reasonably well, it is still unable to effectively deal with the collective behaviors and regular distribution of massive dislocations^12^. Therefore, the aim of this work will be to simplify the evolution process of dislocations by introducing one new concept, to explore in depth the formation mechanism of periodic PSB ladders from a completely new perspective and eventually to build an intuitive bridge between surface slip morphologies and microscopic dislocation patterns.

## Argument

The formation of regular dislocation pattern follows a typical self-organized dissipative process. Firstly, fully reversed cyclic loading produces approximately equal numbers of positive and negative edge-screw mixed dislocations by Frank-read source mechanism^13^. Furthermore, only edge dislocation segments with opposite signs are likely to continuously assemble because unlike screw dislocation segments could easily cross slip and mutually annihilate. The process of mutual trapping of edge dislocation segments continues until achieving an eventual dynamic equilibrium, which is considered to be responsible for saturation within the PSB. Hereafter, the local densities of edge and screw dislocations kept constant. The unit configuration consisting of the mutual capture of edge dislocations is a structure called as “dislocation dipole”. In this process, a large number of dislocations constantly generate, move, interplay, multiply and annihilate; therefore, it will be necessary to simplify the evolution process and find the inner law.

It seems to be a good solution to treat the dislocation motion as a fluctuating process. To prove the existence of the traveling waves representing moving dislocation is an open problem, which is closely related to the nature of wave. In attempt to understand the spatial deeper structures that are concealed in natural light patterns, Nye and Berry^14^ introduced a new concept “wave dislocation” into wave theory: the wavefront can contain dislocation lines, closely analogous to those found in crystals. Following Nye and Berry’s analogy with crystal dislocations, an approach to the Burgers vector of a wave dislocation is proposed by Dennis^15^. It is defined to be a regularized phase gradient evaluated at the phase singularity.

**Figure 1:**
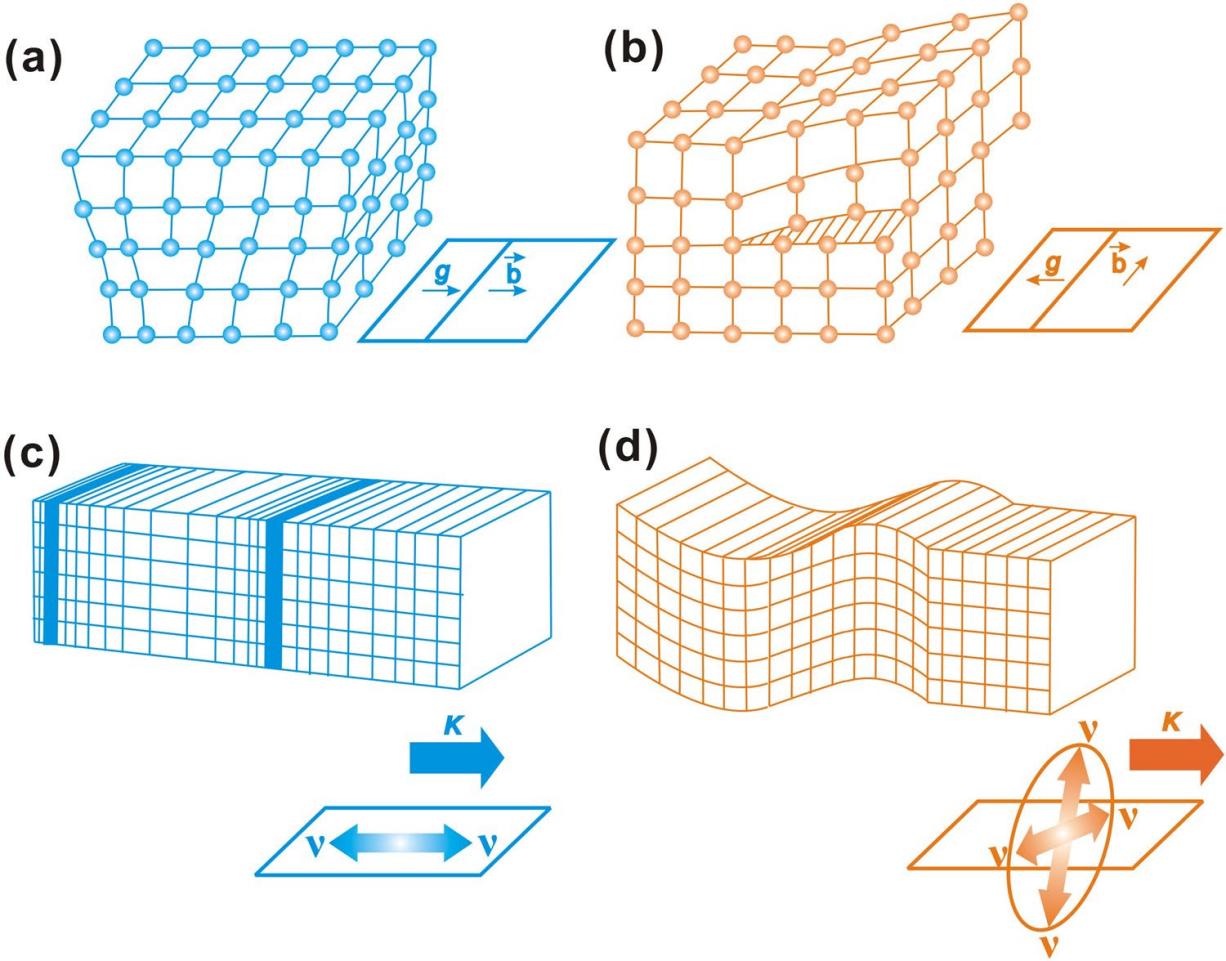
Crystal dislocation and wave. (**a**) edge and (**b**) screw dislocations in crystal lattices, with the arrows showing the dislocation glide direction *g* and Burgers vector *b*, respectively; (**c**) P- and (**d**) S-waves, with the propagation direction *k* and particle vibration direction *v* given by the arrows, respectively.

Hereby, dislocations are recognized as a general phenomenon in scalar wavefields. The Burgers vector in a crystal is more analogous to the phase circulation of ±2π around a wave dislocation. On one hand, a dislocation in a crystal lattice is defined by its Burgers vector, the two most distinctive types are respectively an edge dislocation, whose Burgers vector is perpendicular to the direction of the dislocation line, and a screw dislocation, whose Burgers vector is parallel to the line’s direction, as shown in Fig. 1(a) and (b), respectively. On the other hand, in elastic theory, a dislocation is described as stress singularities of different strength, and for wavefield, phase singularity is the strength. Therefore, by comparing the relative motion between the propagation direction and particle vibration, it could be well judged about the one-to-one relationship between different kinds of dislocations and waves. As shown in Fig. 1(c) and (d), elastic body wave could be divided into P wave and S wave. P waves are also known as compression waves, because particles in the solid vibrate along the direction of wave propagation. However, in the S wave, the medium has particles that vibrate in a direction perpendicular to the direction of wave propagation. Likewise, in edge dislocation, Burgers vector representing atomic motion is parallel to the dislocation glide direction, but the Burgers vector of screw dislocation is perpendicular to the direction of dislocation glide. Specially noted, the velocity of P wave is greater than that of S wave. Similarly, the motion of edge dislocation is faster than that of screw dislocation.

The above elementary arguments have proved that dislocation motion can be considered as elastic wave propagation. The new term “dislocation wave” will simultaneously contain both an elastic wave and a dislocation in each wavefront. The edge dislocation is corresponding to the longitudinal wave and the screw dislocation is equivalent to the shear wave. Since the moving dislocation is a wave, the interaction between dislocations should meet the characteristics of the wave. Therefore, we try to understand the formation of dislocation patterns from the perspective of wave, which will involve one unique concept “standing wave effect”. In physics, the standing wave in a transmission line is a wave in which the distribution of current, voltage, or field strength is formed by the superposition of two waves of the same frequency propagating in opposite directions. The effect is a series of nodes and anti-nodes at fixed points along the transmission line. Such a standing wave may be formed when a wave is transmitted into one end of a transmission line and is reflected from the other end by an impedance mismatch.

## Discussion

**Figure 2:**
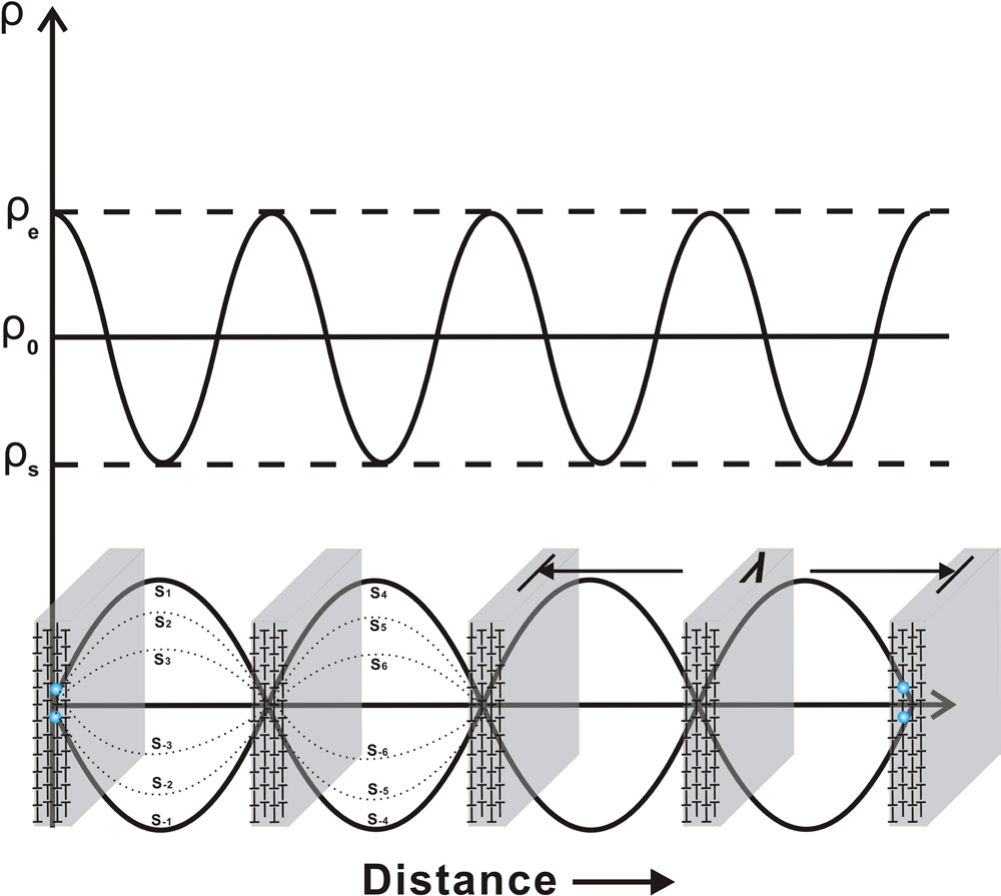
Formation mechanisms of periodic PSB ladders induced by standing wave effect in dislocation evolution.

In the formation of PSB ladders, the cyclic reverse loading likewise induces the standing wave phenomenon. As shown in Fig. 2, in a loading cycle, all the dislocation sources will emit the dislocations by the same frequency, wavelength and wave velocity (S_1_ to S_6_). The mutual capture of unlike edge dislocations at the nodes results from the perfectly timed interference of two waves passing through the same medium. Therefore, the dislocation pattern can be referred to as the standing wave pattern. In the following cycles, the above process is continuously strengthened and more dislocations are added to the node. When a stable dynamic balance is reached, the PSB ladders are formed. The transformation between the screw and edge dislocations represents the constant conversion of energy. The screw and edge dislocations ﻿themselves﻿ denote the kinetic and potential energy, respectively.

Based on the above analysis, the wavelength *λ* of dislocation wave traveling at constant speed is closely related with the distribution of dislocation density. Firstly, a single general law for dislocation multiplication is given by ref. 16

1 $${\dot{\rho }}_{multi}={\rho }_{0}{\dot{\gamma }}_{P}.$$

Here ${\dot{\gamma }}_{P}$ is the resolved plastic strain-rate, *ρ*_0_ denotes the average dislocation density as well as possible dislocation sources within PSBs, expressed by the formula ${\rho }_{0}\approx \sqrt{{\rho }_{e}{\rho }_{s}}$, where *ρ*_*e*_ and *ρ*_*s*_ are the densities of edge and screw dislocations, respectively (see Fig. 2). The dislocation multiplication is caused by the latent dislocation sources and resolved plastic strain-rate. Abiding by the famous Alexander-Hassen empirical model^17^, the resolved plastic strain-rate ${\dot{\gamma }}_{P}$ can be expressed as:

2 $${\dot{\gamma }}_{P}=bv{\rho }_{m}.$$

Where *ρ*_*m*_ designates the density of all the moving dislocation, being almost equal to the density of screw dislocations^18^ and *v* their average velocity. Substituting Eq. (2) into Eq. (1), the following formula could be obtained by analogy to the wavelength equation:

3 $$\frac{v}{(\frac{{\dot{\rho }}_{multi}}{{\rho }_{m}})}=\lambda =\frac{1}{\sqrt{{\rho }_{e}}\cdot b\cdot \sqrt{{\rho }_{s}}}.$$

Here the flow stress is govern by screw dislocation density through the Taylor formula $\tau =\alpha Gb\sqrt{{\rho }_{s}}$ ^19^, where *α* ≈ 0.25, and ${\dot{\rho }}_{multi}/{\rho }_{m}$ denotes the one-way average multiplication from the same dislocation source in one cycle, which is equivalent to the propagation frequency of dislocation wave. It can be imagined when two dislocation wave trains frequently meet at the nodes, the screw segments annihilate each other and the edge segments capture mutually to form the periodic ladder structures. According to the standing wave effect, these ladders represent the nodes in the standing wave. For saturated PSB ladders, *ρ*_*e*_ ≈ 10^16^ m^−2^, thereby, Eq. (3) can be simplified as follows based on the Taylor relation:

4 $$\lambda \approx \frac{40\alpha Gb}{\tau }=K\frac{Gb}{\tau }.$$

Where *K* ≈ 10, Eq. (4) is the same as the “similitude principle” equation firstly proposed by Kuhlmann-Wilsdorf ^20^ and later developed by Gόmez-Garcίa *et al*.^9^. The irreversible plastic deformation of crystalline materials is mainly carried by the dislocation motion. These linear defects carry an elementary amount of shear (the Burgers vector) that is usually the smallest translation of the crystal lattice. Looking upon the dislocation motion as fluctuation process, it will greatly simplify the complication of dislocation interaction, which will be beneficial to understand the dislocation distribution and its relation with slip morphologies.

**Figure 3:**
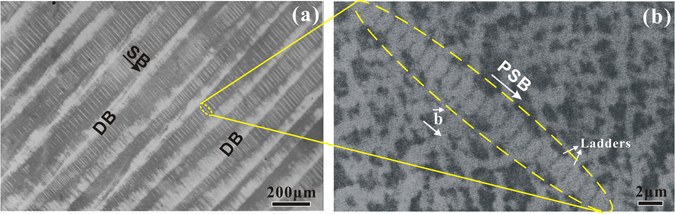
Surface slip morphology and corresponding dislocation patterns of the fatigued Cu single crystal. (**a**) SBs and DBII; (**b**) PSBs.

Besides the general SBs as well as the well-known PSBs, the appearance of DBs seems to be another important feature induced by cyclic deformation. Figure 3(a) shows the classical slip morphologies of cyclically deformed Cu single crystals^21^, which consist of slip bands (SBs) and deformation bands (DBs). In general, two types of DB, namely DBI and DBII, as well as occasionally DBIII, have been identified in fatigued Cu single crystals, where DBI is approximately parallel to the primary slip plane, while DBII makes a certain angle with the primary slip plane^22–26^. These DBs disrupt an initially smooth surface of the specimen, influencing not only the process of initiation of fatigue crack but also their subsequent direction of propagation. As shown in Fig. 3(a), DBII and SBs are roughly perpendicular to each other and the spacing of the DBs is about 100~110 μm. The statistical result shows^27^ that the density of SBs within DBII is twice, even more than the density of SBs in-between DBII. Li *et al*.^28^ have pointed out the interacting angle between the primary SBs and DBII varies with the change of the observed plane. On the best observed plane $(1\bar{2}1)$, DBII and SBs strictly follows the mutual perpendicular crystallographic relationship. Further, Li *et al*.^29^ demonstrated that the appearance of DBII consisting of the wall structures is due to the lacking of the activation of the secondary slip systems. They considered that the formation of the wall within DBII may be derived from the rotation accumulation of geometrical necessary dislocations (GNDs). Likewise, Wang *et al*.^30–32^ found the effect of the dislocation activities on the grain rotation, grain boundary sliding, as well as dislocation interaction in smaller grain by using *in*-*situ* atomic scale straining inside TEM. Therefore, the concept of dislocation wave could be extended to the above field, also including the interactions between dislocation and pore, dislocation and precipitate, etc.

Corresponding to the SBs in-between DBII, Fig. 3(b) exhibits the geometrical structure and fundamental characteristics of PSB. In general, PSB ladders are a 3D structure and occupy about 10% of the PSBs by volume while the ladders are 0.03~0.25 μm in thickness with a spacing of about 1.3 μm. By comparing Fig. 3(a) and (b), it could be found that the distribution of PSB ladders is very similar to those of SBs and DBII. Therefore, it could be concluded that PSB ladders are not only self-organized but also self-similar dislocation structures. What does this self-similar distribution mean?

It is also well known that the fractal is a mathematical set that exhibits a repeating pattern at every scale, which is called as a self-similar pattern. Mandelbrot^29^ pointed out that the fractals also include the idea of a detailed pattern that repeats itself. Fractal analysis provides an important tool to account for multiscale behavior, which will address the important question of how the macroscopic properties of a material relate to its microscopic defect structure^30^, while in practice this becomes a difficult exercise due to the unclear dislocation distribution of the mesoscopic scale, that is how edge dislocations distribute in one PSB ladder based on self-similar principle.

As stated earlier in Fig. 2, based on the standing wave effect, the capture of edge segments at the nodes shows that the dislocation loops are bowed out along both sides of the ladders at equal intervals, thus its interval distance is the same as the ladder thickness *d*_*w*_. Meanwhile, the mean interval distance ${d}_{\perp \perp }$ of edge dislocation in the ladders is approximately 8 times as long as the extended dislocation width *d*_*ex*_^33^. Considering Shockley partials in pairs, substituting ${d}_{w}/2\approx 1/\sqrt{{\rho }_{s}}$ and ${d}_{\perp \perp }/2\approx 1/\sqrt{{\rho }_{e}}$ into Eq. (3), the self-similar relation can be obtained as below,

5 $$\frac{{d}_{w}}{{\lambda }}=\frac{4b}{{d}_{\perp \perp }}.$$

Here *λ* ≈ 2*d*_*c*_, *d*_*c*_ denotes the channel width. Equation (5) could be rewritten as

6 $$f=\frac{{d}_{w}}{{d}_{c}}=\frac{8b}{{d}_{\perp \perp }}\approx \frac{{d}_{ex}}{{d}_{\perp \perp }}={2}^{-3}.$$

**Figure 4:**
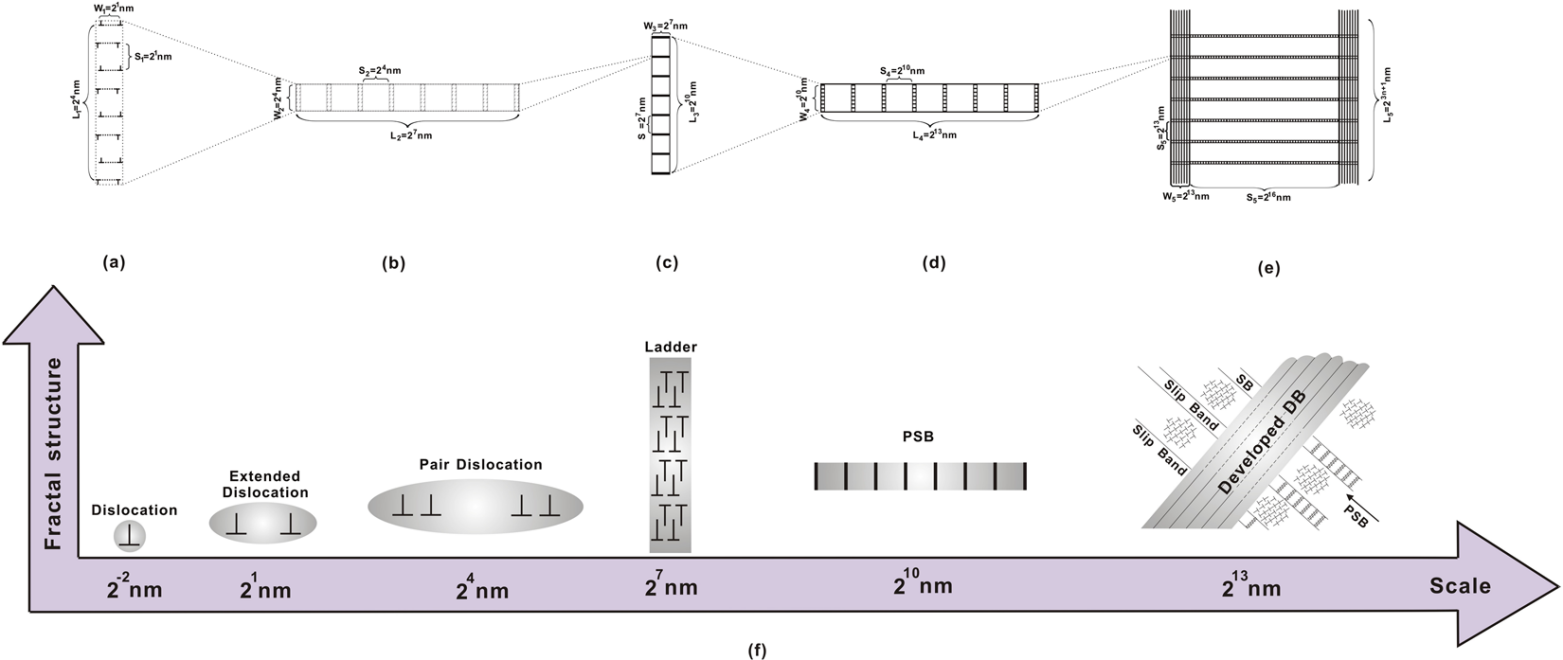
Dislocation Distribution based on the fractal principle. (**a**–**e**) Self-similarity of dislocation distribution; (**f**) Dislocation structural parameters at different scales.

Where *f* is defined as the self-similar factor. Equation (6) fully demonstrates the self-similarity and fractal feature of dislocation distribution. From dislocation dipole to DBs, Fig. 4 describes the fractal distribution of edge dislocations at different scales in details. Firstly, according to Eq. (6), several important distribution parameters will be listed: *W*, *S* and *L* denote the width, spacing and length of self-similar structure, and the edge dislocations distribute in terms of the relation *W* = *S* = *f L*. In one dipole, *W*_1_ and *S*_1_ are the extended width and the trap distance of dislocations, respectively, which is equal to 2^1^ nm (see Fig. 4(a)). Subsequently, the fractal structure will constantly repeat itself to distribute and fill in the whole deformed crystalline material, as shown in Fig. 4(b–e). During this group of self-similar structures, several key parameters are identified and summarized in Fig. 4(f), where *S*_2_ = 2^4^ nm represents the spacing distance of dislocation, *W*_3_ = 2^7^ nm is the ladder thickness, *S*_4_ = 2^10^ nm denotes the channel width as well as SB width and *W*_5_ = 2^13^ nm is the DB width. Based on the above construction details, the fractal dimension will be introduced to analyze the dislocation distribution more quantitatively.

The fractal dimension is characterized as a measure of the space-filling capacity of a pattern, firstly discussed by Mandelbrot^34^, and is usually not an integer. Hausdorff ^35^ uses the magnification factor *r* and the number of self-similar objects *N* to calculate the fractal dimension, $D=\mathrm{log}\,{N}/\mathrm{log}\,r$. By analyzing the self-similar structures at different scales in Fig. 4, the fractal dimension of dislocation evolution can be written as below:

7 $$D=\,\mathrm{log}\,8/{\mathrm{log}(1/2}^{-3})=1.$$

Equation (7) demonstrates that the dislocations distribution is in accordance with one-dimension linear and the forming pattern is self-similar to its minimum defect–one limited stacking fault (see Fig. 4(a)), which is the reason that the crack induced by the fractal distribution of dislocations will initiate and propagate along the SBs or DBs (see Fig. 4(e)).

It should be noted that the present evidences only obtain from SB, PSB and DB in the fatigued FCC metals. There is still no direct evidence for the fracture structure. Considering that the dislocation wave represents a possible way of dislocation movement, it will be only corresponding to the elastic-plastic deformation stage and is not currently suitable for the final fracture stage. Here, the fractal dimension characterizes the intrinsic trend of the failure fracture of crystals. Recently, Li *et al*.^36^ explained reasonably the external reason that leads to the preferential nucleation of cracks within DBs by comparing respective carried plastic shear strains of DBs and PSBs. The combination of internal and external factors will completely interpret the plastic deformation and fracture behaviors of fatigued FCC crystals.

In summary, dislocation motion can be regarded as elastic wave propagation. The new term “dislocation wave” will simultaneously contain both an elastic wave and a dislocation in each wavefront. The edge dislocation is corresponding to the longitudinal wave and the screw dislocation is equivalent to the shear wave. Based upon the standing wave effect, the formation of periodic PSB ladders can be attributed to the annihilation of screw dislocation segments and mutual capture of edge dislocation segments at the nodes. Not only self-organized but self-similar dislocation pattern distributes based on the fractal principle. The fractal dimension reveals the intrinsic nature of crack initiation and propagation along SBs and DBs.
